# Impact of honey on dental erosion and adhesion of early bacterial colonizers

**DOI:** 10.1038/s41598-018-29188-x

**Published:** 2018-07-19

**Authors:** Alexandra Habluetzel, Christoph Schmid, Thiago S. Carvalho, Adrian Lussi, Sigrun Eick

**Affiliations:** 1 Department of Restorative, Preventive and Pediatric Dentistry, School of Dental Medicine, University of Bern, Bern, Switzerland; 2 Department of Periodontology, School of Dental Medicine, University of Bern, Bern, Switzerland

## Abstract

The aim was to investigate if honey causes erosion and if salivary pellicle modified with honey, or its components, or the by-product propolis has a protective effect against dental erosion and adhesion of early bacterial colonizers. The tested substances were: 3 types of honey, methylglyoxal (MGO), hydrogen peroxide, propolis. First in the erosion experiment, 120 human enamel specimens were covered with salivary pellicle and modified with the substances. Then they were eroded with 1% citric acid, pH 3.6 for 2 min, before surface hardness was measured. In the microbiological assay, the enamel specimens (n = 126) covered with modified salivary pellicle were contaminated with bacterial suspensions. The antimicrobial activity of each substance and their effect on early bacterial colonizer adhesion and biofilm formation were determined. Despite a low pH, honey did not cause erosion. On the other hand, pellicle modification with the tested solutions did not protect the enamel from erosion. Microbiologically, the 3 honeys inhibited species-specific growth of oral bacteria. Propolis decreased initial attachment of *Streptococcus gordonii*, while one honey inhibited demineralization of enamel by biofilm. In conclusion, pellicle modification with honey, or its components, or propolis did neither protect against erosion nor promote it. Propolis presented some bacterial adhesion inhibition.

## Introduction

Both dental erosion and caries derive from a demineralization of the dental hard tissue, but these conditions have different aetiologies. Erosion is a result of a chemical process without the involvement of bacteria, whereas the metabolic products of bacteria cause caries. The acquired enamel pellicle (AEP) has a protective effect against both caries and dental erosion^1–5^ and the modification of the AEP, e.g. by casein and mucin, seems to show great promise in prevention of erosion and bacterial colonization^6,7^.

Other potential candidates for modifying the pellicle are ingredients of natural products or plant extracts with polyphenolic contents^8^. Non-toxic and inexpensive substances are good choices to modify the pellicle, for example substances like tannic acid, Inula viscosa tea, and safflower oil^8–11^. Honey is one of these natural products of interest, for it contains many polyphenols^12^.

Honey has been known to treat infected wounds for thousands of years^13^. Nowadays honey regains more and more attention because of its broad-spectrum antimicrobial effects, with favourable results even against *Staphylococcus aureus*^14–17^. Its antimicrobial activity is generally linked to several components, but mainly bee defensin, polyphenols or hydrogen peroxide (H_2_O_2_), the latter being a major biomarker for the antibacterial activity in several kinds of honey^18^. However, some kinds of honey do not have this H_2_O_2_ activity. These so-called non-peroxide activity honeys have a high concentration of MGO, which is responsible for their antimicrobial activities^19^. These kinds of honey, for example the Manuka honey^17,20^, have sparked much interest in research.

Manuka honey is collected from flowering *Leptospermum scoparium* (manuka) plants in New Zealand^17,20^. Its antimicrobial activity is mostly related to MGO, one of its major components^21,22^. In Dentistry, Manuka honey has been tested in a pilot study, where patients were asked to use a Manuka honey product during 21 days, and they presented a significant reduction in plaque score and gingivitis^23^.

Propolis is also an interesting substance with antimicrobial effect^24^. Similarly to honey, it is rich in phenolic compounds: flavonoid aglycones (flavones and flavanones), phenolic acids, and their esters. In medical use, propolis has been tested for cutaneous wound healing, as well as an antimicrobial agent in oral hygiene, such as in toothpaste and mouthrinse^25–27^. Since propolis and honey have high phenolic contents, we have aimed at testing their capability to modify the AEP, and verify their effect on dental erosion and bacterial adhesion. Furthermore, since the antimicrobial activity in honey generally comes from H_2_O_2_, and that in Manuka honey comes from MGO, both these compounds were also tested separately. Therefore, the present study aimed at answering the following questions:

- Do different kinds of honey, H_2_O_2_, MGO, or propolis cause erosion?
- Does a pellicle modified with different kinds of honey, H_2_O_2_, MGO, or propolis protect enamel against hardness loss when attacked by acids?
- Do different kinds of honey, H_2_O_2_, MGO, or propolis inhibit the growth of cariogenic bacteria and bacterial attachment to enamel surfaces?

Our hypotheses were that the different kinds of honey, H_2_O_2_, MGO, or propolis are not erosive to dental enamel surface and the pellicle modification with these products would have a protective effect against acids and cariogenic bacteria.

## Material and Methods

The present study was made up of two experiments, one was an erosion experiment, and another was a microbiological assay. For both parts, we used the same tested products for pellicle modification; whole mouth stimulated human saliva (HS) for pellicle formation; and enamel specimens from human teeth.

### Tested products and their chemical properties

A total of 8 groups were included in the study: 3 types of honey, 2 components of honey, propolis, and 2 control groups:

- The three kinds of honey were: a Swiss midland honey (Gallmann; Forschungsanstalt Agroscope Liebefeld-Posieux ALP Zentrum für Bienenforschung ZBF, Switzerland), a German lowland honey (provided by a local beekeeper, Wolmirstedt, Germany) and a Manuka honey (MGO^TM^250 + Manuka Honey, New Zealand). To use in pellicle modification, the honeys were diluted 1:1 in HS.
- Two components of honey were used: Methylglyoxal (MGO, 40% solution in H_2_0, Sigma-Aldrich Buchs, Switzerland) was diluted in deionized water to obtain a final solution of 10% MGO. The pH of the MGO solution was later set to 5.5. Another solution of 2 mM hydrogen peroxide (H_2_O_2_) was also used (Pharmacia, Inselspital Bern, Switzerland).
- Propolis was originally obtained as a 50% tincture in ethanol (Apinatura, Naters, Switzerland). The tincture was then diluted in deionized water to obtain a 10% propolis and 10% ethanol solution.
- As controls, we had two groups. A non-modified salivary pellicle was one control. Since the propolis contained ethanol, we included a 10% ethanol solution in addition.

For the chemical properties of the solutions, we analysed their pH and calcium concentrations. The pH of all test products was determined with a pH electrode connected to Metrohm pH-meter 691 (Herisau, Switzerland). Calcium concentration was analysed with a flame atomic adsorption spectrometer (AAnalyst 400, Perkin Elmer Analytical Instruments, Waltham, Massachusetts, USA) equipped with acetylene-air gas input. Lanthanum nitrate (4.2%, aqueous solution) was used in the atomic adsorption analysis of calcium to hinder the interference of phosphate ions on the detection signal.

### Saliva collection

The whole mouth stimulated human saliva (HS) was collected from healthy volunteers (aged 18–40). Because the saliva was pooled, the ethics committee categorizes it as “irreversibly anonymised”, and therefore no previous ethical approval from the the local ethics committee (Kantonale Ethikkommission: KEK) was necessary. Saliva collection was carried out in accordance with the approved guidelines and regulations of the KEK. The volunteers were informed about the use of their saliva in research, and their oral consent was obtained. For saliva collection, the volunteers chewed on a piece of paraffin (Fluka; Sigma-Aldrich Chemie GmbH, Munich, Germany) for 10 min, and HS was collected in chilled flasks maintained in ice. HS was then pooled, centrifuged for 15 min at 4 °C and 400 g and stored at −80 °C until the time of experiment. For the microbiological assay, the saliva was exposed to UV radiation for 30 min to become sterile.

### Preparation of enamel specimens

A total of 246 enamel specimens were prepared from human molars previously stored in a pool of extracted teeth. Because the teeth are from a pool of extracted teeth, the local ethics committee (KEK) categorized them as “irreversibly anonymised”, and therefore no ethical approval was necessary. The teeth were extracted by dental practitioners in Switzerland (no water fluoridation, 250 ppm F^−^ in table salt) and were stored in 2% chloramine T trihydrate solution. The patients were informed about the use of their teeth and oral consent was obtained. These specimens were then divided into two major groups; one part was used in the experiments focusing on erosion (n = 120) and the other part (n = 126) in the microbiological assay. The specimens were prepared by separating the crowns from the roots, and then cutting the crowns in a mesio-distal direction with the IsoMet® Low speed Saw (Buehler, Düsseldorf, Germany), creating buccal and lingual halves. Each half was embedded in resin (Paladur, Heraeus Kulzer GmbH, Hanau, Germany) and serially abraded under constant tap water cooling using Knuth Rotor machine (LabPol 21, Struers, Copenhagen, Denmark) with silicon carbide paper discs of grain size 18.3 μm, 8 μm and 5 μm, 60 seconds each. The enamel specimens were then polished for 60 seconds with 3 mm abrasive diamond polishing cloths on Struers under constant cooling after been taken out of the moulds (LaboPol-21, DP-Mol Polishing, DP-Stick HQ, Struers, Copenhagen, Denmark). Between and after all polishing levels, the specimens were sonicated for 1 min.

### Erosion Experiment

#### Pellicle formation and modification

The 120 enamel specimens were divided into 8 groups (n = 15/group). Pellicle formation was made by incubating the specimens in 1.5 ml HS for 2 h at 37 °C in a shaking water bath (70 rpm). The specimens were then washed in deionized water, dried, and submitted to pellicle modification. Pellicle modification was made by incubating the specimens in 1.5 ml of the respective solution (Table 1) at 37 °C in a shaking water bath (70 rpm). The pellicle modification was made for a total of 2, 4 and 30 min. The negative control (non-modified pellicle) group was incubated in a humid chamber in the same conditions as the other groups. After each time lapse, as well as at baseline, surface hardness was measured.

**Table 1:** pH and calcium concentration in the substances used in the experiment.

Substance	pH before dilution	pH after dilution	Ca (mM)
**Saliva**			
HS used for pellicle formation	7.7	—	1.08
**Test Products**			
Swiss midland honey (SMH) diluted in HS (1:1)	4.6	5.3	1.09
Manuka honey diluted in HS (1:1)	4.1	5.4	1.44
German lowland honey (GLH) diluted in HS (1:1)	3.7	5.8	1.74
2 mM H_2_O_2_	5.9	8.0	—
10% MGO solution	5.5*	5.8	<0.1
10% Propolis in 10% ethanol	4.6	7.6	<0.1
10% ethanol solution (control)	6.1	8.2	<0.1

HS = Whole mouth stimulated human saliva;

*Adjusted pH of the MGO solution. The original 40% MGO solution had pH of 2.3 and 0.11 mM calcium.

#### Erosion challenge

After 30 min of pellicle modification, the specimens were submitted to an erosive challenge, by incubating them for 2 min in 1% citric acid (pH 3.6; 25 °C, under constant agitation 70 rpm). The specimens were then removed from the acid, rinsed with deionised water for 20 s, and dried with air for 5 s, and surface hardness was measured again.

### Microbiological Assay

#### Bacterial strains

The bacteria used in the experiments were *Streptococcus gordonii* ATCC 10558, *S. sanguinis* ATCC 10556, *S. mutans* ATCC 25175, *S. sobrinus* ATCC 12104, *Lactobacillus acidophilus* ATCC 11975 and *Actinomyces naeslundii* ATCC 12104. Before the tests, all bacterial strains were passaged on tryptic soy agar (Oxoid, Basingstoke, GB) with 5% sheep blood with 10% of CO_2_ at 37 °C for 24 h.

#### Determination of minimal inhibitory concentration

To measure the minimal inhibitory concentrations (MICs) of the test substances against the different bacterial strains, each substance was serially diluted two-fold in microtiter plates. Bacterial suspension (McFarland 0.5) of the tested strains was mixed 1: 9 with nutrient media (Wilkins Chalgren broth (Oxoid), lysed horse blood (5%), β-NAD (2%) and added to the microtiter plates. After 24 hours of incubation with 10% CO_2_, the MICs (turbidities) were determined with a spectrophotometer at OD 600. To confirm the results, suspension of each well was subcultured on Schaedler agar plates.

#### Bacterial adhesion assay

The 126 enamel specimens used for the microbiological assay were sterilized at 121 °C in a humid atmosphere for 10 min before they were finally polished on the polishing cloth. In the bacterial adhesion assay only one control group (saliva only) was used, as the 10% ethanol solution did not exert any antimicrobial activity in the MIC assays. Two different bacterial adhesion assays were made. In one, only *S. gordonii* ATCC 10558 was included as an early colonizer; in the second, we used a mixed “cariogenic” population consisting of *S. sanguinis* ATCC 10556, *S. mutans* ATCC 25175, *S. sobrinus* ATCC 12104, *Lactobacillus acidophilus* ATCC 11975 and *Actinomyces naeslundii* ATCC 12104. To form a pellicle layer, enamel specimens were coated with 50 µl of 30 min UV exposed sterile human saliva at room temperature for 2 h. After this time the remaining saliva was carefully removed from the enamel surface (the samples were kept wet) and the enamel specimens were soaked in 50 µl of the test substance mixed 1:1 with saliva for 30 min at 37 °C. Both control groups were incubated in a humid chamber in the same conditions as the other groups. Thereafter, the remains of substances were sucked off with a vacuum suction pump and the enamel specimens were exposed to UV for 30 min.

In the single-species experiments, 42 enamel specimens (6 per group and 3 for the controls) were then contaminated with 10^8^/ml *S. gordonii* ATCC 10558 suspension for 30 min and 2 h in DMEM (Dulbecco’s modified Eagle’s medium; Gibco, Invitrogen). After a short dipping into 0.9% w/v NaCl to remove no adhering bacteria, the adhered bacteria were removed by intensive wiping the enamel surface with a cotton swab. The swabs were transferred into 1 ml of 0.9% w/v NaCl. Aliquotes were given on agar plates and after 48 h of incubation with 10% CO2 at 37 °C the colony forming units (cfu) were enumerated.

In the second series, after pellicle modification, 84 enamel specimens (2 per assay with 6 specimens and 3 control) were contaminated with 1 ml suspension consisting of each 10^8^/ml *S. mutans* ATCC 25175, *L. acidophilus* ATCC 11975, *S. sobrinus* ATCC 12104 and *A. naeslundii* ATCC 12104. To verify also a long-term exposure to sucrose, brain-heart infusion broth (BHI, Oxoid, Basingstoke, GB) with 5% sucrose was applied for 4 h and exchanged by BHI broth containing phosphate buffer for 17 h. The procedure was repeated twice. After 24 h and 48 h since the beginning of the experiment, each specimen was taken out of the medium cleaned with a soft toothbrush (Trisa Vitaclean soft) for 10 s with a moderate pressure. Then, pellicle was formed and modified again as before and after adding nutrient media, bacteria were supplemented, too.

After 72 h the bacterial counts (cfu) were assessed as described before, however the incubation time of the agar plates was 96 h.

In addition, before and after the experiment, the surface hardness of the enamel specimens was measured.

### Surface hardness measurements

For both erosion experiment and microbiological assay, we measured surface hardness of the enamel specimens with a Vickers diamond under 50mN pressure for 15 s (Fisherscope HM 2000 XYp; Helmut Fischer, Hünenberg, Switzerland). The specimens were always placed in the same position on the device for baseline and further measurements. For each SH measurement, six indentations were made at intervals of 50 μm from each other and the average value from the six indentations was considered as SH value for the specimen. The change in SH between the initial measurement (at baseline) and the following measurements were calculated as a percentage (∆%SH) and used for statistical data analysis and interpretation. ∆%SH was calculated using the formula: ∆%SH = (SH_i_/SH_0_) × 100, where SH_0_ is the initial hardness value and SH_i_ is the hardness value of the i^th^ measurement (the i^th^ incubation in the pellicle modification solution, or after the erosion, or after incubation in biofilm).

### Statistical analysis

Statistical analysis was made with SPSS 24.0 (IBM Corporation, New York, NY, USA). First% of change of surface roughness was calculated. Results were related to baseline as 100%. The data showed a non-normal distribution, so between-group comparisons at the respective time-points were made with Kruskal-Wallis test and the Mann-Whitney U-test was applied for comparison of single compounds with the respective control. The level of significance was set at p < 0.05.

## Results

### PH and Ca content of the test materials

The pH and Ca content of the test substances are presented in Table 1. The table shows that, after dilution in HS, the pH increases.

### Erosion experiment

**Figure 1:**
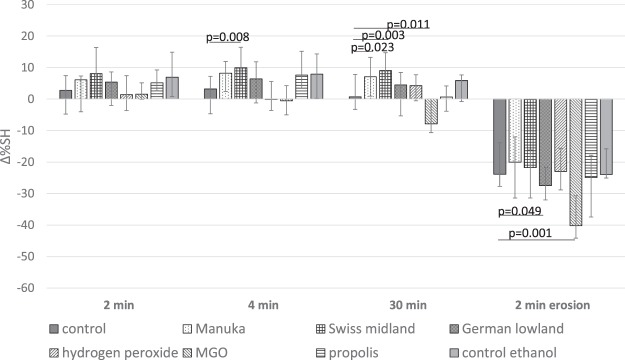
Change in surface hardness (∆%SH; median incl. 25 and 75 percentiles) related to baseline after pellicle modification for 2, 4, or 30 min, and after 2 min erosion in citric acid, according to the test groups. NOTE: Negative values represent decrease in SH, positive values represent increase in SH. Statistically significant differences between the modification and the respective control are shown.

The change in SH after pellicle modification and erosion are presented in Fig. 1. There were statistically significant differences between groups at 4 min (p = 0.001), at 30 min of incubation (p < 0.001) related to baseline. Comparing to the control group (non-modified pellicle), Manuka honey increased surface hardness at 30 min (p = 0.023) and Swiss midland honey at 4 min (p = 0.008) and 30 min (p = 0.003) of pellicle modification, whereas MGO decreased surface hardness even without erosion challenge at 30 min (p = 0.011).

Furthermore, after exposing the enamel surface to citric acid, there was also a statistically significant difference between the groups (p < 0.001), where the erosive challenge caused a decrease in enamel surface hardness. In comparison to the control group (non-modified pellicle), pellicle modification with the German lowland honey (p = 0.049) and the MGO (p = 0.001) caused greater decrease in surface hardness.

### Microbiological assay

The minimum inhibitory concentrations (MIC) values for the tested substances are presented in Table 2. Highly concentrated honey inhibited the growth of selected oral microorganisms. However, bacteria most associated with caries were inhibited only at very high concentrations whereas the MICs of the honeys were lower against commensals. Manuka honey, in comparison with the other honeys, was more growth inhibitory at lower concentration against four of the six tested bacterial species. Both MGO and propolis were active against the bacterial species in concentrations of ≤0.31% and 1.25% respectively. Hydrogen peroxide did not inhibit bacterial growth up to the tested concentration of 2 mM (Table 2). The positive control 10% ethanol solution did not exert any antimicrobial activity with any of the strains.

**Table 2:** Minimum inhibitory concentration (MIC) values for the different honeys, their components and propolis against selected oral species.

	Manuka honey	Swiss midland honey	German lowland honey	Hydrogen peroxide	MGO in solution	Propolis in 10% ethanol*
*S. gordonii* ATCC 10558	1.25%	1.25%	1.25%	>2 mM	≤0.31%	1.25%
*S. sanguinis* ATCC 10556	1.25%	1.25%	1.25%	>2 mM	≤0.31%	1.25%
*S. mutans* ATCC 25175	20%	>50%	>50%	>2 mM	≤0.31%	1.25%
*S. sobrinus* ATCC 33478	10%	50%	50%	>2 mM	≤0.31%	1.25%
*A. naeslundi*i ATCC 12104	5%	10%	1.25%	>2 mM	≤0.31%	≤0.63%
*L. acidophilus* ATCC 11975	1.25%	50%	2.5%	>2 mM	≤0.31%	≤0.63%

*10% ethanol did not exert any antimicrobial activity.

**Figure 2:**
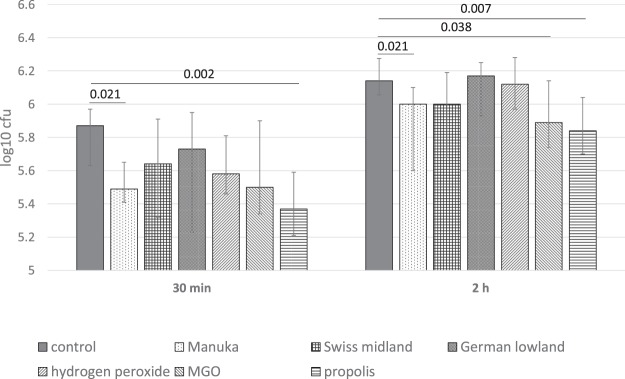
Adherence of *S. gordonii* ATCC 10558 (cfu; median incl. 25 and 75 percentiles) to enamel surfaces after pellicle modification with the different substances. Statistically significant differences between the modification and the control are shown.

Regarding the adhesion of *S. gordonii*, an early colonizer, we observed statistically significant differences between groups at 30 min (p = 0.046) and at 2 h (p = 0.007). Modification with propolis and with Manuka honey reduced the adherence of *S. gordonii* ATCC 10558 in comparison with the control after 30 min (p = 0.002, p = 0.021) and 2 h (p = 0.007, p = 0.021). MGO acted also anti-adhesive, however the difference was only statistically significantly after 2 h (p = 0.038; Fig. 2).

**Figure 3:**
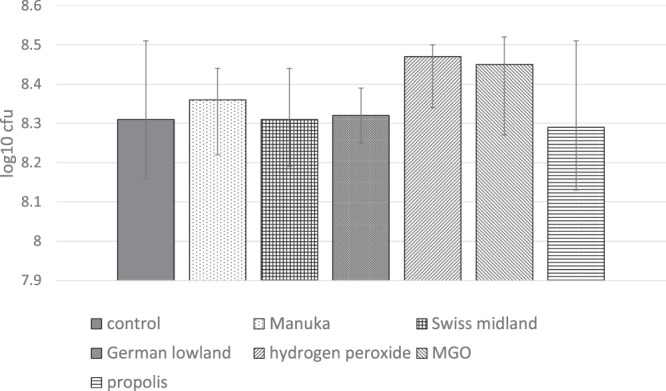
Total bacterial count (cfu, median incl. 25 and 75 percentiles) attached to enamel surface after pellicle modification and after 72 h of biofilm formation and intermitted “cleaning”. Statistically significant differences between the modification and the control are shown.

The assays with the six-species biofilms did not result in any differences in the bacterial counts in comparison with the control after daily intermitted “brushing” cleaning for three days (Fig. 3).

**Figure 4:**
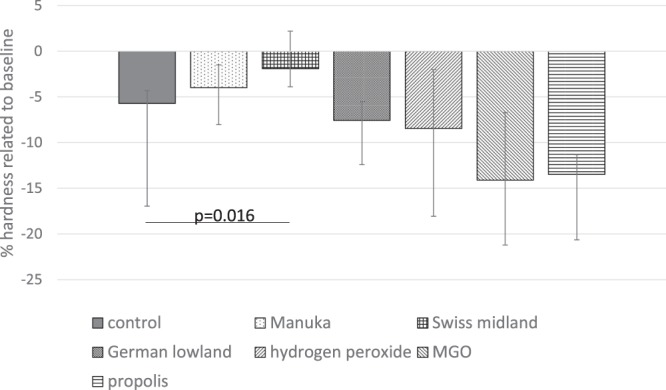
Enamel surface hardness change (∆%SH; median incl. 25 and 75 percentiles) related to baseline after pellicle modification and after 72 h of biofilm formation and intermitted “cleaning”. NOTE: Negative values represent decrease in SH, positive values represent increase in SH. Statistically significant differences between the modification and the control are shown.

After incubation in biofilm with intermittent cleaning, SH was also analysed. The difference between all groups was statistically significant (p = 0.006). The result of the Swiss midland honey showed nearly no difference in SH (−1.90% in median), whereas there was a higher change of SH (−8.32% in median) in the non-modified pellicle (p = 0.016). The other modifications decreased the SH in median by 3.98% (Manuka honey) up to 14.1% (MGO), however the differences were not statistically significant differences when compared with the control (Fig. 4).

## Discussion

Honey, its ingredients, and propolis have been thought as preventive agents against caries and gingivitis^28–30^. Since some of these substances also have a low pH, this *in vitro* study investigated if they cause dental erosion. We also investigated if these substances are able to modify the enamel acquired pellicle (AEP) leading to a protective effect against erosion and cariogenic bacteria. Our results show that the MGO was the only substance to cause some dental erosion after 30 min incubation, but none of the honeys, nor the other components, nor propolis caused dental erosion. Moreover, when they were used to modify the AEP, they were also not able to prevent enamel dissolution when exposed to citric acid as an erosive challenge. Interestingly, pellicle modification with the Swiss midland honey was protective against cariogenic surface hardness loss.

For our material and methods standardized conditions were provided. The enamel specimens were prepared as in earlier investigation^7^, and we measured Vickers hardness to detect the enamel hardness loss, as has been used in other similar studies^31,32^. Our microbiological assay adhesion model used sterilized human saliva to precede the microbial adhesion on the enamel surface^33,34^, and the intermitted cleaning of the surface by using a soft tooth brush should have led to an *in vitro* representation of tooth brushing in the oral environment^35^.

In this study, we used the tested honeys diluted 1:1 in HS. Because of their high viscosity, the honeys needed to be diluted to allow their use in the experiment. On the other hand, diluting the honeys with human saliva could have already caused a reaction between the active ingredients from the honeys and the proteins in the saliva. This could have led to a reduced modification of the pellicle, and a reduction in any possible protective effect that could be expected from the honeys. However, the other tested solutions (H_2_O_2_, MGO and propolis) that were not diluted in HS could have greater chances at modifying the pellicle in this protocol.

Initially, we tested whether the tested substances were erosive. The three different tested honeys as well as the other tested substances were detected as being acidic. Even after dilution of the three honeys with saliva, the pH of the substance was always below pH 5.8. From previous studies it is well known, that pH is one of the main indicators for dental erosion; if pH of the tested substance decreases, more erosion can be measured^36^ and a low pH at the enamel surface may lead to mineral loss^37^. Despite the low pH of the three different tested honeys, no erosive activity on the enamel surface was detected. This is in agreement with Grobler and collaborators, who showed that honey, despite its low pH, did not cause erosion after 30 min in contact with teeth^37^.

It is well known that not only the pH but also the calcium (Ca^2+^) concentration in the substances is of importance regarding erosion^38–40^. Ca^2+^ can reduce or decrease the extent of demineralisation, as explained by the Law of Mass Action^41^. A supersaturation of Ca^2+^ is well-known as a protective factor^42^. In our experiments, the Ca^2+^-contents of the honeys ranged from 1.09–1.74 mM. Similar Ca^2+^-content has also been observed in vitamin C (1.78 mM)^43^, which, due to the low pH (3.6), is significantly associated with dental erosion^44^. It was remarkable that the German lowland honey with a similar pH and Ca^2+^-content to the vitamin C, did not cause erosion even after 30 min incubation. This “anti-erosion” effect of the honeys should be investigated further. Furthermore, it is not possible to pinpoint which kind of honey would best modify the AEP, for different kinds of honey will have different calcium concentrations. In this study, we have chosen three kinds of honey to observe their specific effects on the AEP. However, every kind of honey has its own composition, such as different proteins and mineral ions, which can influence the modification of the AEP, so direct comparisons should be made with caution.

Despite the fact that honeys did not cause dental erosion, the modification of the AEP with the honeys did not lead to further protection against subsequent erosion with citric acid. In our experimental protocol, one should consider the fact that the three tested honeys were diluted in HS 1:1 before the tests. In such a case, the Ca^2+^ in the honey could react with salivary proteins within the HS^4^ still during the dilution process, thus leading to less build-up of modified pellicle. Likewise, the polyphenols present in the honey could have undergone the same process, thus reducing any possible protective effect after pellicle modification.

Honey has a notoriously high sugar content, and the microbial assay in this study was necessary for an adequate overview on the effects of this high sugar content on early colonizers. The three honeys have shown different minimal inhibitory concentrations against the selected oral bacterial strains (*Streptococcus gordonii ATCC 10558, S. sanguinis ATCC 10556, S. mutans ATCC 25175, S. sobrinus ATCC 12104, Lactobacillus acidophilus ATCC 11975 and Actinomyces naeslundii ATCC 12104*).

Manuka honey, compared to the other honeys, inhibited the growth of the selected oral species at a lower concentration. This high antibacterial activity of Manuka honey was as expected^45^. Previous studies already showed that Medical-grade Manuka honey acts antimicrobial towards representative oral bacteria in general, and the gram-negative anaerobes associated with gingivitis are particularly sensitive^46^. Manuka honey showed also superiority when tested against methicillin-resistant *Staphylococcus aureus*^16^.

However, it should be mentioned that the antibacterial activity of Manuka honey seemed to be species-specific. *S. mutans ATCC 25175* was less sensitive against Manuka honey comparing the other tested oral species. Similar results were shown before. Schmidlin and co-workers found different Manuka honey preparations more active in inhibiting the growth of *P. gingival is* and *A. actinomycetemcomitans* rather than those of *S. mutans*^45^.

Propolis initially inhibited adhesion of *S. gordonii ATCC 10558* to the pellicle when compared to the control. An antibacterial activity of propolis was already demonstrated in several studies^47,48^. Propolis interferes with enzyme activity and cell division, thus resulting in antimicrobial properties^49^. When inhibiting *S. gordonii* ATCC 10558, an early colonizer it can be suggested that propolis might retard the bacterial adhesion on early biofilm formation.

First interactions of bacteria on the tooth surface can result from sedimentation, active movement of the bacteria, diffusion and liquid flow. Electrostatic, hydrophobic and van der Waals forces can lead to nonspecific, reversible adhesion of the bacteria to the pellicle on the surface^50^. Specific stereochemical interactions between bacterial adhesions and complementary receptors in the acquired pellicle can lead to an irreversible attachment^51^.

When using a multi-species biofilm model, no significant influence of any of the potential test substances was found. This behaviour underlines that surface modifications interfere only initially with biofilm formation.

Also nearly no difference in enamel hardness loss was seen when modified pellicle was exposed to multi-species biofilm. The intermitted cleaning procedure might influence that finding. Tooth cleaning and an efficient oral hygiene were found to have a caries preventive effect. Studies have shown that the quality of tooth cleaning is more effective than the quantity of its performance^35^.

Strikingly, the presence of honey in the pellicle, despite its high sugar content, did not promote enamel demineralization even after incubation in a cariogenic biofilm. In this study, microbial biofilm resulted in less hardness loss when the pellicle was modified with Swiss Midland honey in comparison with propolis. A previous study showed that the pH recovers much faster after honey than sugar exposures and never did drop below the critical decalcification pH of 5.5 for enamel caries^28^. Moreover, it is important to bear in mind that this critical pH is only for cariogenic demineralisation of enamel, and there is no fixed critical pH value concerning dental erosion^2^.

In conclusion, the honeys and the by-product propolis did not cause enamel erosion, but the methylglyoxal (MGO) did after 30 min incubation. Although the enamel pellicle modification with honey, its ingredients and the by-product propolis did not protect the enamel from further erosive challenges, it did not favour the growth and adhesion of cariogenic bacteria.
